# Towards rare earth element recovery from wastewaters: biosorption using phototrophic organisms

**DOI:** 10.1007/s00253-021-11386-9

**Published:** 2021-06-18

**Authors:** Marcus Heilmann, Roman Breiter, Anna Maria Becker

**Affiliations:** Institute of Bioprocess Engineering, Department of Chemical and Biological Engineering, Faculty of Engineering, Friedrich-Alexander-Universität Erlangen-Nürnberg, Paul-Gordan-Straße 3, 91052 Erlangen, Germany

**Keywords:** Gold, Microalgae, Moss, REE, Sorption, Wastewaters

## Abstract

**Abstract:**

Whilst the biosorption of metal ions by phototrophic (micro)organisms has been demonstrated in earlier and more recent research, the isolation of rare earth elements (REEs) from highly dilute aqueous solutions with this type of biomass remains largely unexplored. Therefore, the selective binding abilities of two microalgae (*Calothrix brevissima*, *Chlorella kessleri*) and one moss (*Physcomitrella patens*) were examined using Neodym and Europium as examples. The biomass of *P. patens* showed the highest sorption capacities for both REEs (Nd^3+^: 0.74 ± 0.05 mmol*g^−1^; Eu^3+^: 0.48 ± 0.05 mmol*g^−1^). A comparison with the sorption of precious metals (Au^3+^, Pt^4+^) and typical metal ions contained in wastewaters (Pb^2+^, Fe^2+^, Cu^2+^, Ni^2+^), which might compete for binding sites, revealed that the sorption capacities for Au^3+^ (1.59 ± 0.07 mmol*g^−1^) and Pb^2+^ (0.83 ± 0.02 mmol*g^−1^) are even higher. Although different patterns of maximum sorption capacities for the tested metal ions were observed for the microalgae, they too showed the highest affinities for Au^3+^, Pb^2+^, and Nd^3+^. Nd-sorption experiments in the pH range from 1 to 6 and the recorded adsorption isotherms for this element showed that the biomass of *P. patens* has favourable properties as biosorbent compared to the microalgae investigated here. Whilst the cultivation mode did not influence the sorption capacities for the target elements of the two algal species, it had a great impact on the properties of the moss. Thus, further studies are necessary to develop effective biosorption processes for the recovery of REEs from alternative and so far unexploited sources.

**Key points:**

*• The highest binding capacity for selected REEs was registered for P. patens.*

*• The highest biosorption was found for Au and the biomass of the examined moss.*

*• Biosorption capacities of P. patens seem to depend on the cultivation mode.*

## Introduction

Rare earth elements (REEs), consisting of lanthanum (La), the lanthanides (Ce, Pr, Nd, Pm, Sm, Eu, Gd, Tb, Dy, Ho, Er, Tm, Yb, Lu) plus yttrium (Y) and scandium (Sc), are important for modern, high-tech devices and applications such as smartphones, solar cells, electric vehicles and power generation (Guyonnet et al. 2015; Ambaye et al. 2020). A global demand of 160,000 t of REEs (as their oxides) was estimated for 2016 (Hatch 2012). The growing demand for high-tech products requires an increasing availability of resources and, considering the urgent need for environmentally friendly industrial solutions, calls for sustainable production and adequate recycling. At the same time, due to the random distribution of REEs in the earth’s crust, their ecological production seems to be a challenge. For example, ion-adsorbing clays, which are the main natural source of REEs, reach at best REEs contents of 0.1 weight % (Mariano & Mariano Jr. 2012). Therefore, their exploitation is associated with tremendous amounts of waste rock and in addition provides hazardous materials such as toxic metals, acids, fluorides and radioactive material (Öko-Institut 2011; Humphries 2013). Besides that, due to the risk of supply shortage and their impact on the economy, REEs were assigned by the European Commission to the list of 27 critical raw materials (European Commission Communication 2017); the USA added REEs to the list of 35 critical minerals in 2018 (US Department of Interior 2018).

In the search for potential novel REE production technologies, the sorption ability of biomass was found to be promising (Heilmann et al. 2015). Investigations of metal binding from aqueous solutions for example by algal biomass are not new; Kuyucak and Volesky (1988) reviewed early approaches for the treatment of industrial waste and process waters. So far, most developments involved microalgae for heavy metal recovery studies, where metal ions from wastewaters were removed from solutions via biosorption without additional desorbing steps (Veglio and Beolchini 1997; Wilke et al. 2006). However, biosorption is also considered one of the very promising biological methods for the recovery of metals from electronic waste, as it is very efficient and cost-effective and avoids the generation of chemical sludge (Ambaye et al. 2020; Giese 2020).

Microalgae and other phototrophic organisms, which can be cultivated in open or closed bioreactor systems, have lately received great attention due to their diversity and number of technologically interesting compounds or properties. They are not only considered a source of various native metabolites that find their use in food industry, such as natural pigments and antioxidants. Their biomass can also be used for extraction of dyes, such as malachite green or methylene blue, from wastewater (Khataee et al. 2013; Vijaraghavan et al. 2015; Ruangsomboon et al. 2013; Gupta et al. 2014).

This work focuses on biosorption abilities and binding mechanisms of selected biological species regarding REEs. In order to utilise so far unexploited and/or unconventional sources of REEs, such as mining drainage waters, seepage waters from mine dumps and mining, process waters, electrical wastes after chemical digestion and highly diluted REE solutions were particularly considered sources of the target elements in this study. Hence, the aim of this work was to characterise the sorption of selected REE representatives (Neodym and Europium) on the biomass of three biological species: *Chlorella kessleri* (green alga), *Calothrix brevissima* (cyanobacterium) and *Physcomitrella patens* (moss) that were identified in our previous work as promising biosorbent candidates (Heilmann et al. 2015). Sorption experiments under various conditions, such as at different pH values and REE concentrations (sorption isotherms), were conducted, and sorption capacities for other precious metals (Au^3+^, Pt^4+^) as well as for metal ions typically contained in wastewaters (Pb^2+^, Fe^2+^, Cu^2+^, Ni^2+^) were determined in order to evaluate the suitability of the biomass for REE recovery from different aqueous solutions.

## Materials and methods

### Instrumentation, chemicals and organisms

Quantification of elements from aqueous solution was performed with either by spectrophotometric assay—based on the colour reaction with xylenol orange (XO) as described by Heilmann et al. (2015) and a multilabel reader (EnSpire 2300, Perkin Elmer, USA; for concentrations ≥ 100 μM of Nd^3+^ and Eu^3+^)—or by inductively coupled plasma atomic emission spectroscopy (ICP-AES, CIROS CCD, Spectro, Germany; for concentrations of Nd^3+^/Eu^3+^ ≤ 100 μM and quantification of Au^3+^, Pb^2+^, Nd^3+^, Pt^4+^, Eu^3+^, Fe^2+^, Cu^2+^ and Ni^2+^). For pH measurements, a pH-metre (pH 510, EUTECH Instruments, Germany/SevenGo, Mettler Toledo, USA) in combination with a microelectrode (InLab/InLab Micro, Mettler Toledo, USA) was used. All chemicals used in this study were of analytical grade and were purchased from either Fluka, Carl Roth, Merck (Germany) or Sigma-Aldrich (USA). Chemical elements for biosorption experiments were used as aqueous solutions of their chlorides (AuHCl_4_, PtCl_4_), sulphates (CuSO_4_, FeSO_4_, NiSO_4_) or nitrates (Eu(NO_3_)_3_, Nd(NO_3_)_3_, Pb(NO_3_)_2_). Stock solutions of target elements had a concentration of 10 mM. *Chlorella kessleri* SAG 211-11g and *C. brevissima* SAG 34.79 were obtained from the SAG - Culture Collection of Algae at Göttingen University (Germany) and *P. patens* IMSC 40001 from the IMSC - International Moss Stock Centre at Freiburg University (Germany). The organisms were cultivated in shake flasks (0.1-L scale), bubble columns (1-L scale) and in an airlift reactor (15-L scale) as described in the “Cultivation of biospecies” section.

### Cultivation of biospecies

Table 1 gives an overview of the cultivation conditions at the different scales. Shake flask cultivation was performed under axenic conditions for up to 14 days. For the axenic biomass production at the 1-L scale, a shake flask culture was used for inoculation and the bubble column was operated for further 14 days under constant external illumination. In the case of *P. patens*, also, the biomass obtained on the 15-L scale in an internally illuminated airlift photobioreactor was used (Heining et al. 2015; Heining 2016).

**Table 1:** Cultivation conditions used in this study

Cultivation vessel	Scale	Medium	Conditions
Shake flask	0.1 L	M1^a)^	Illumination: external, 100 μmol*m^−2^*s^−1^ Temperature: RT Shaking: 40 rpm, 5 cm orbital motion
Bubble column	1 L	M1^a)^	Illumination: external, 80 μmol*m^−2^*s^−1^ Temperature: 25 °C Aeration: 0.5 L*min^−1^ air enriched with 3 vol.-% CO_2_
Airlift reactor	15 L	Modified Knop^b)^	Illumination: wireless light emitters (WLE)^a)^ 60 μmol*m^−2^*s^−1^ Temperature: 25 °C Aeration: 0.5 L*min^−1^ air enriched with 1 vol.-% CO_2_

^a)^Heining et al. (2015)

^b)^Heining (2016)

Whereas biomass from shake flask cultures was employed for the determination of sorption capacities from single element solutions of Nd^3+^ and Eu^3+^ (as described in the “Cultivation of biospecies” section), all other experiments were performed with the biomass obtained from the cultivation in bubble columns (*C. kessleri*, *C. brevissima*) or in the airlift photobiorector (*P. patens*).

### Determination of biosorption capacity of the selected species

The biomass of *P. patens*, *C. kessleri* and *C. brevissima* was lyophilised, grounded using mortar and pestle and stored at −20 °C. For sorption experiments, biomass was weighed and placed in empty reaction tubes, stirred with a magnetic stirrer for 1 h in distilled water (2 mL) and centrifuged (10 min, 10,000×*g*, room temperature), and the supernatant was removed. The weight of the wet biomass was recorded to determine water residue, resulting from the biomass swelling, to correct the metal ion concentrations. Various experiments were performed to investigate the adsorption behaviour of the selected biological species regarding different (target and disturbing) elements. All biosorption capacity experiments were performed in triplicates with at least one to two repetitions on different days.

First, maximum sorption capacities (Q_max_) of *P. patens*, *C. kessleri* and *C. brevissima* obtained from shake flask cultivation for two representatives of the REEs (Nd^3+^/Eu^3+^) from single element solutions were examined. Therefore, selected biomass, prepared as described above, was placed in 2 mL of a 10 mM solution of either REE (pH = 5) to reach biomass concentrations of 5 to 20 g*L^−1^ and stirred with a magnetic stirrer for 24 h at room temperature. Various amounts of biomass were examined to appoint the maximum sorption capacity for each species. The samples were centrifuged (10 min, 10 000×*g*, room temperature) and the metal ion concentrations in the supernatant analysed via XO assay as described by Heilmann et al. (2015). Each sample was measured three times and sorption capacities (Q) were calculated using Eq. 1:

1 $$ Q=\frac{n_i-{n}_f}{m} $$

where n_i_ corresponds to the initial, n_f_ to the final amount of the metal ion in the supernatant and m refers to the dry weight of the biomass used in the experiment.

Next, maximum biosorption capacities (Q_max_) for Nd^3+^_,_ Eu^3+^_,_ Au^3+^, Pt^4+^, Fe^2+^, Cu^2+^ and Ni^2+^ from single element solutions (10 mM) were determined, according to the procedure described above, using the biomass of *C. kessleri* and *C. brevissima* cultivated in bubble columns and of *P. patens* from the 15-L airlift reactor. These experiments were done to characterise the biosorption properties of the named species regarding the selected metal ions, to estimate the affinity of possibly competing metal ions to the selected biosorbers and to compare the sorption of REEs on the biomass resulting from different cultivation systems.

### Influence of the pH value on the sorption capacity of the biomass

To investigate the influence of acidic conditions during biosorption, sorption capacities of lyophilised biomass of *C. brevissima, C. kessleri* and *P. patens* (10 mg respectively) for Nd^3+^ in the pH range from 1 to 6 were determined in triplicates and with at least one to two repetitions on different days. For this purpose, the biomass was prepared as described above and incubated with the respective Nd solution under stirring for 3 h. The pH was monitored and kept constant during the experiment by adding 0.1 M NaOH/HCl, and each experiment was performed in triplicates. The acid/base volume added to the Nd^3+^ solution was registered for the correction of the metal concentration in the supernatant, which was obtained either via XO assay (Heilman et al. 2015) (for pH 3 to 6) or via ICP-AES (for pH 1 and 2). The equilibrium sorption capacity (Q_eq_) was calculated according to Eq. 1.

### Adsorption isotherms

For the evaluation of the sorption behaviour of a biomass at different Nd^3+^concentrations, the biomass of all three appointed species (10 mg corresponding to 5 g_biomass_*L^−1^) was lyophylised and washed as described under the “Cultivation of biospecies” section was incubated for 3 h with concentrations of Nd^3+^ ranging from 0.5 to 6.5 mM (n = 3). The pH during the incubation was kept constant at 5. After the incubation, the biomass suspension was centrifuged (10 min, 10,000×*g*, room temperature), the Nd^3+^ concentration in the supernatant determined using XO assay (Heilman et al. 2015) and the Q_eq_ was calculated according to Eq. 1.

### Acid/base-titrations of biomass

Acid/base titration curves for *C. brevissima*, *C. kessleri*, and *P. patens* were prepared with biomass that was washed (as described above) and resuspended in 1 mM NaCl solution (10 mL). For this purpose, 25 to 475 μL of either HCl or NaOH (0.1 M) was added and the pH of the supernatant was registered after exactly 2 h of stirring. As reference, the pH values of the 1 mM NaCl solution after addition of the same amounts of HCl/NaOH were measured, and all obtained points plotted against the corresponding volume of acid/base. Finally, based on the resulting curves, the proton exchange capacity (PEC) and pK_a_ values were calculated for each biomass with ProtoFit 2.1 (Turner and Fein 2006).

## Results

### Sorption capacity of the selected species

**Fig. 1:**
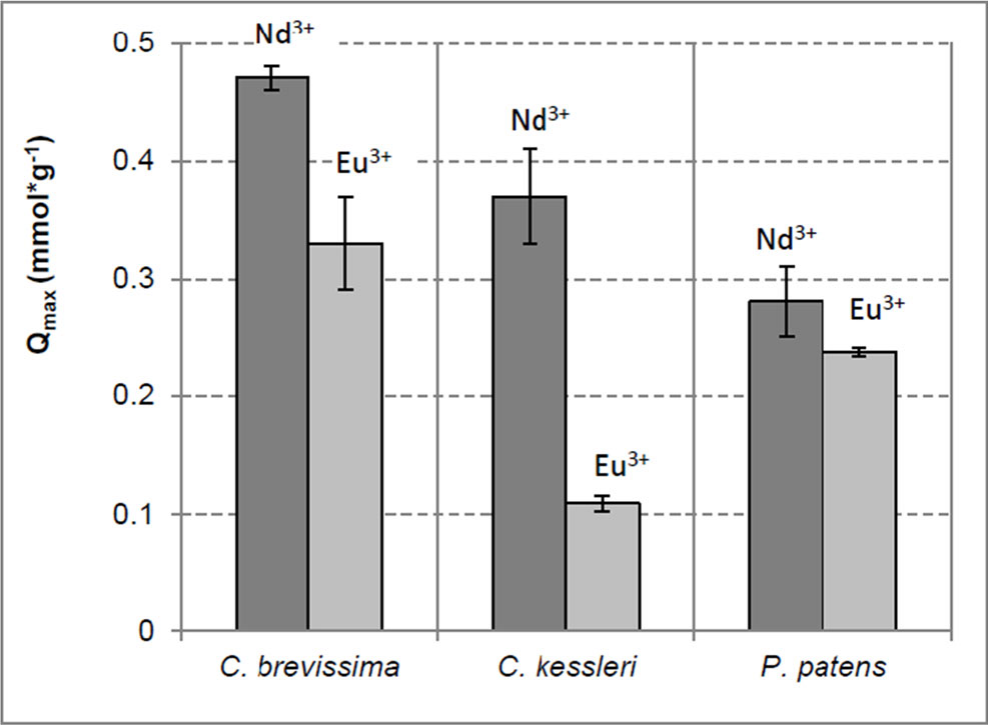
Maximum sorption capacity of *C. brevissima*, *C. kessleri* and *P. patens* biomass from externally illuminated shake flask cultivation*.* Sorption experiment (24 h) performed with 5 to 20 g*L^−1^ dried biomass and 10 mM initial concentration of Nd^3+^/Eu^3+^ at room temperature and initial pH of 5 (n = 3). Dark grey bars correspond to Q_max_ of Nd^3+^, light grey bars to Q_max_ of Eu^3+^

First, maximum biosorption capacities (Q_max_) of the species that were found to be promising candidates for biosorption of REEs in our previous study (Heilmann et al. 2015) were examined. Therefore, *C. brevissima*, *C. kessleri* and *P. patens* were cultivated under similar conditions in shake flasks. Collected, lyophilised and washed biomass was incubated with Nd^3+^/Eu^3+^ for 24 h. The results are depicted in Fig. 1. Each of the three examined species showed a higher Q_max_ for Nd^3+^ than that for Eu^3+^. The highest Q_max Nd_ was registered for *C. brevissima* with 0.47 ± 0.01 mmol*g^−1^, followed by *C. kessleri* (0.37 ± 0.04 mmol*g^−1^) and *P. patens* (0.28 ± 0.03 mmol*g^−1^). A slightly other order was found for the second examined REE with the highest Q_max Eu_ again for *C. brevissima* (0.33 ± 0.04 mmol*g^−1^), followed by *P. patens* (0.24 ± 0.01 mmol*g^−1^) and *C. kessleri* (0.11 ± 0.01 mmol*g^−1^).

**Fig. 2:**
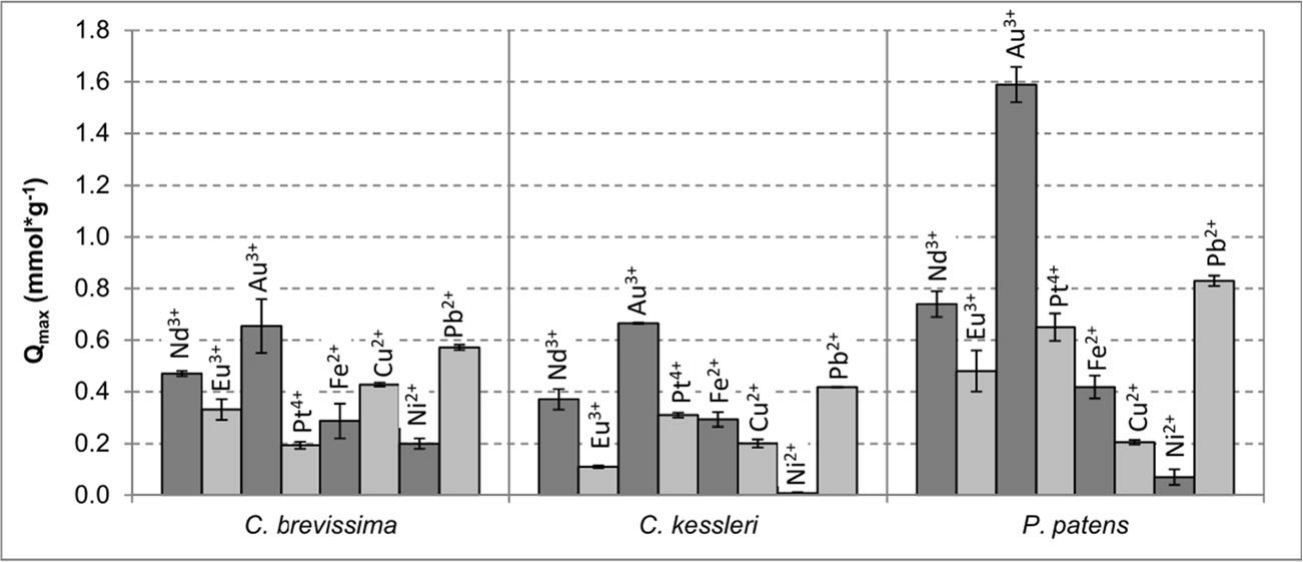
Sorption capacities of *C. brevissima*, *C. kessleri* and *P. patens* for different metal ions from single aqueous element solutions of the target ion. Sorption experiment (24 h) performed with 5 g*L^−1^ of dried biomass and 10 mM initial concentration of respective metal ions at room temperature and initial pH of 5 (n = 3). The biomass of *P. patens* was obtained from an internally illuminated airlift culture (Heining et al. 2015); those of *C. brevissima* and *C. kessleri* were generated in externally illuminated bubble columns

Next, to evaluate the affinities of the selected biological species to various metal ions, Q_max_ values with single element solutions of Au^3+^, Pb^2+^, Nd^3+^, Pt^4+^, Eu^3+^, Fe^2+^, Cu^2+^ and Ni^2+^ were determined for biomasses of all three organisms from photobioreactors (Fig. 2). Of all ions, the highest Q_max_ value was measured for Au and *P. patens* (1.59 ± 0.07 mmol*g^−1^). *Calothrix brevissima* and *C. kessleri* as well showed the highest affinity for this metal ion resulting in Q_max_ of 0.66 ± 0.01 mmol*g^−1^ and 0.65 ± 0.1 mmol*g^−1^, respectively. The second highest binding capacity was observed for Pb ranging from 0.42 ± 0.01 mmol*g^−1^ for C. *kessleri* to 0.83 ± 0.02 mmol*g^−1^ for *P. patens*. The third highest value was registered for Nd and again for all three biomasses examined here with Q_max_ from 0.37 ± 0.04 mmol*g^−1^ (*C. kessleri*) up to 0.74 ± 0.05 mmol*g^−1^ (*P. patens*). The lowest Q_max_ values of all ions were determined for Ni, ranging from 0.008 ± 0.004 mmol*g^−1^ for *C. kessleri* to 0.2 ± 0.02 mmol*g^−1^ for *C. brevissima*. Concerning the investigated biomasses, *P. patens* showed binding capacities that were up to 2.4 times higher (Au) than the ones of the other two species. Most of the other metal ions tested here were also bound more effectively by *P. patens* than by either of the two remaining biosorbents, with the exception of Cu and Ni. Surprisingly, the sorption capacities of *P. patens* for Nd^3+^ and Eu^3+^ in this second experiment were 2.6-fold and twofold higher than in the first experiment. The obvious difference between these two experiments was the method of biomass production suggesting that the moss biomass characteristics differed significantly between shake flask and WLE airlift reactor culture. A comparable discrepancy was not observed for the algal/cyanobacterial biomass from shake flasks and bubble columns.

### Characterisation of the biosorption of REEs by the biomass of *C. brevissima*, *C. kessleri* and *P. patens*

#### Adsorption isotherms at constant pH

For the intended applications, such as biosorption from wastewater and seepage water, high sorption capacities already at low concentrations of the respective ions are desirable. Thus, sorption isotherms must be considered to select a suitable biosorber material. For this reason, the sorption of Nd^3+^ at various initial concentrations at room temperature and constant pH of the solution (pH = 5) by the biomass of *C. brevissima*, *C. kessleri* and *P. patens* was registered. The resulting adsorption isotherms were fitted with functions described by Langmuir (1932) or Freundlich (1906) that are described by equations given below (Eqs. 2 and 3).

Equation 2 Langmuir’s isotherm with Q = sorption capacity; Q_max_ = maximum sorption capacity; c = concentration of Nd^3+^; b = specific constant, depending on temperature and adsorption enthalpy.

2 $$ Q=\frac{Q_{max}\ast c}{b+c} $$

Equation 3 Freundlich’s isotherm with Q = sorption capacity; c = concentration of Nd^3+^; a, m = specific constants, depending on the system.

3 $$ Q=a\ast {c}^{1/m} $$

**Fig. 3:**
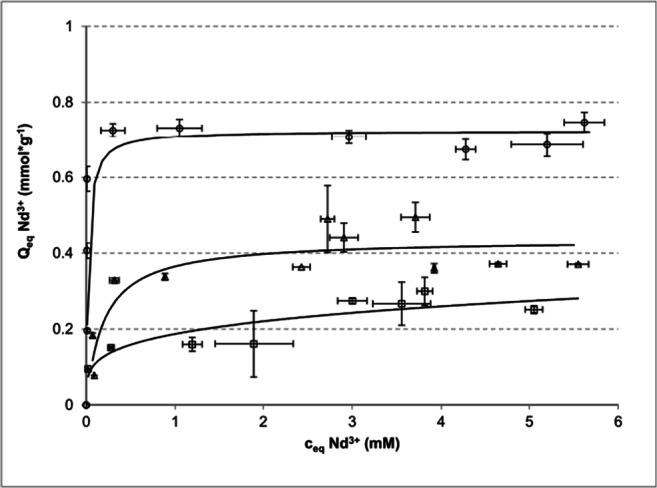
Sorption isotherms recorded at constant pH of the Nd^3+^-solution for *C. brevissima* (triangles), *C. kessleri* (squares) and *P. patens* (circles). Nd^3+^ solutions of initial concentrations ranging from 0.5 to 6.5 mM were incubated (room temperature, 3 h, pH 5) with dried biomass of the investigated species (10 mg corresponding to 5 g_biomass_*L^−1^) (n = 3). The biomass of *C. brevissima* and *C. kessleri* was cultivated in bubble columns, whereas *P. patens* was obtained from cultivation in a WLE airlift reactor (Heining et al. 2015)

As the pH can be crucial in evaluating adequate sorption capacities, adsorption isotherms were recorded at a strictly defined pH value of 5, close to the original pH of the Nd^3+^ stock solution (10 mM, pH = 5.2). Nd^3+^ adsorption isotherm curves are presented in Fig. 3. The isotherm for *P. patens* showed Langmuir-like behaviour, displaying a very steep slope at low Nd equilibrium concentrations (0.0095–0.3 mM) and reaching its maximum at 0.75 mmol*g^−1^ already around 0.3 mM of Nd^3+^, which corresponds very well with the Q_max_ of Nd^3+^ obtained with the same biomass and described above (the “Sorption capacity of the selected species” section). The isotherm of *C. brevissima* could also be fitted well to the Langmuir function and showed a steep initial slope reaching Q_max_ of 0.49 ± 0.09 mmol*g^−1^, also being in a good agreement with the maximum sorption capacities obtained in both previous experiments. In contrary, the adsorption isotherm for Nd^3+^ and *C. kessleri* could be better fitted with Freundlich’s isotherm.

#### Biosorption from solutions of various pH

**Fig. 4:**
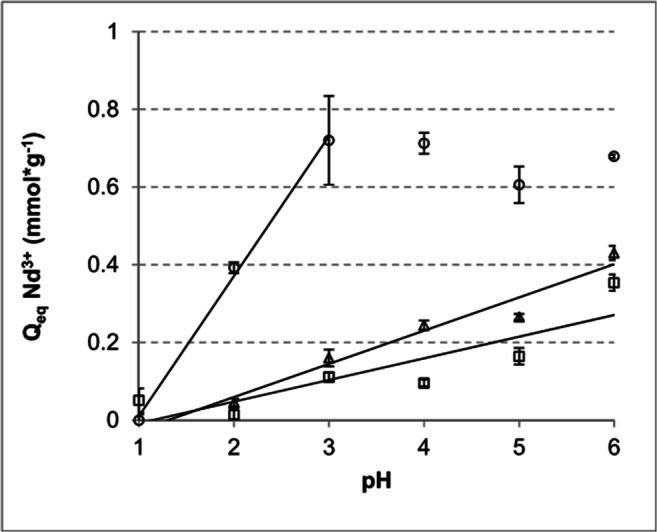
Equilibrium sorption capacities for Nd^3+^ (Q_eq, Nd_) at different pH values (pH 1–pH 6) of a Nd solution for *C. brevissima* (triangles), *C. kessleri* (squares) and *P. patens* (circles). Lyophied biomass of the three species (10–30 g*L^−1^) was incubated with Nd-solutions for 3 h at room temperature and washed in distilled water prior to the sorption experiments (n = 3); *C. brevissima* and *C. kessleri* - PSM cultures, *P. patens* - WLE airlift culture (Heining et al. 2015)

Due to precipitation and sedimentation of most REE hydroxides at values above pH 7, increased metal ion concentrations are to be expected particularly in acidic wastewater samples. Therefore, the biosorption of Nd^3+^ at different pH values ranging from 1 to 6 was examined for the biomasses of *C. brevissima*, *C. kessleri* and *P. patens* (Fig. 4). As depicted in Fig. 4, expectedly for all tested biomasses, the Q_eq, Nd_ increased with increasing pH, showing however different initial slopes. Whereas the sorption capacities determined for *P. patens* in this experiment reached their maximum already around pH of 3 (0.72 ± 0.03 mmol*g^−1^) and remained nearly constant up to pH 6, positive, linear correlations between Q_eq, Nd_ and increasing pH values were observed for *C. brevissima* and *C. kessleri* with the highest sorption values reached only at pH 6 (0.43 ± 0.14 mmol*g^−1^; 0.35 ± 0.14 mmol*g^−1^, respectively), again corresponding well with the previously obtained data.

#### Proton exchange capacity of the selected species

**Fig. 5:**
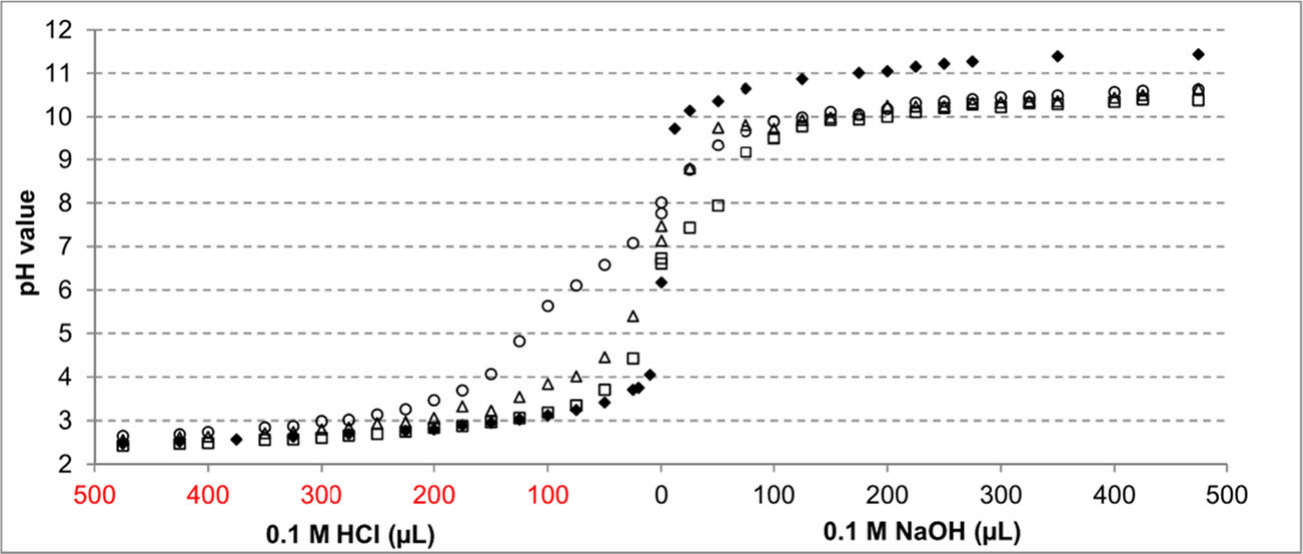
Acid-base titration curves of dry biomass of *C. brevissima* (triangles), *C. kessleri* (squares) and *P. patens* (circles) and without biomass (diamonds). Base/acid (0.1 M NaOH/0.1 M HCl) was added to dried and with ultrapure water washed biomass (2 mL, 1 h, room temperature, mixing) of the selected species (10 mg) suspended in 10 mL of 1 mM NaCl; pH values were measured 2 h after addition of titration agent (n = 1); *C. brevissima* and *C. kessleri* - bubble column cultures, *P. patens* - WLE airlift culture (Heining et al. 2015)

To evaluate the proton exchange capacity (PEC) and to help to assess the binding mechanism of REE sorption on the biomass, acid-base titration studies were performed with the lyophilised biomass of *P. patens*, *C. kessleri* and *C. brevissima*. The biomass was washed with deionised water and resuspended in NaCl solution; hence, the protonation grade at the starting point of titration remained unchanged. The titration curves of the dried biomass of these three species are shown in Fig. 5. The protonation grades of the examined biomasses were calculated based on the difference between the pH of the reference (without biomass) and the pH of the biomass suspension prior to the base/acid addition. The respective PEC values were calculated based on the titration endpoints and the resulting pK_a_ values using ProtoFit 2.1 (Turner and Fein 2006). Chemical properties calculated from titration data (Fig. 5) are shown in Table 2. The highest PEC was calculated for the biomass of *P. patens* (3.48 mmol*g^−1^), followed by *C. brevissima* (2.95 mmol*g^−1^) and *C. kessleri* (2.51 mmol*g^−1^) with protonation grades of 67, 79 and 100 %, respectively. As the actual pK_a_ value of a functional group depends on the location in the macromolecular structure and chemical surrounding, definite classifications of the functional groups involved in sorption were not possible. On the other hand, it is highly probable that carboxy groups were found in all the species tested with pK_a_ values ranging from 4.2 (*C. brevissima*) to 5.5 (*P. patens*), indicating terminal carboxyl groups of peptides and glycoproteins. Hydroxy groups could be determined as more acidic ones, e.g., terminal hydroxyl groups in glycoproteins (pK_a_ 6.3 and 8.3) and more basic ones in phenolic side chains (pK_a_ 10.4 to 11.3). Moreover, pK_a_ values around 11 suggest α-amino groups and pK_a_ values of 6.3 phosphate groups.

**Table 2:** Chemical properties of biomass binding sites, determined by titration of dry biomass and calculated via ProtoFit 2.1

	*C. brevissima*^a)^	*C. kessleri*^a)^	*P. patens*^b)^	Functional group
PEC (mmol*g^−1^)^c)^	2.95	2.51	3.48	-
Protonation rate (%)	79	100	67	-
Q_max, Nd_ (mmol*g^−1^)	0.47	0.37	0.75	-
Q_max, Eu_ (mmol*g^−1^)	0.33	< LOD^d)^	0.48	-
pK_a_1	4.2	4.8	4.3	Carboxy group
pK_a_2	11.3	10.4	11.0	α-Amino group/phenolic OH
pK_a_3	/	6.3	6.3	Phosphate/hydroxy group
pK_a_4	/	8.3	5.5	Carboxy group/hydroxy group

^a)^Bubble column culture

^b)^WLE airlift reactor culture (Heining 2016)

^c)^*PEC* proton exchange capacity

^d)^*LOD* limit of detection

## Discussion

### Sorption capacity of the selected species

The highest sorption capacities from a single ion metal solution of the selected REE representatives (Nd^3+^/Eu^3+^) were found in this study for the moss biomass of *P. patens* reaching up to 0.75 mmol*g^−1^ for Nd^3+^ and 0.48 mmol*g^−1^ for Eu^3+^. This Nd value corresponds well with the Q_max, Nd_ reported in our previous study (Heilmann et al. 2015). In both cases, the results were obtained using biomass that was cultivated in an innovative internally illuminated photobioreactor (Heining et al. 2015). Surprisingly, the biomass of the same moss raised in the identical culture medium but in shake flasks with external illumination showed a Q_max_ of only 0.28 ± 0.03 mmol*g^−1^ for Nd^3+^ and 0.24 ± 0.01 mmol*g^−1^ for Eu^3+^. The latter capacities were thus even lower than those found for the two microalgae examined here. The biomass of *C. brevissima* and *C. kessleri* produced in shake flasks and in bubble columns showed very similar sorption capacities for Nd^3+^/Eu^3+^ of about 0.5/0.3 mmol*g^−1^ for the first and 0.4/0.1 mmol*g^−1^ for the second species. Thus, these data suggest that the cultivation mode does not strongly influence the biosorption properties of either the cyanobacterium or of the green alga, but rather those of the moss species — *P. patens*. The higher sorption capacity of the moss biomass from WLE airlift cultivation might possibly be explained by an increased biomass surface and thus more exposed binding sites, due to the specific lightning mode delivered by the WLE and by the continuous mechanical stress during this cultivation.

Independent from the experimental setup, all three examined biological species can bind Nd more effectively than Eu. Although only few reports are available on sorption of REEs on phototrophic species, making comparisons quite difficult, some data for example for bacteria, yeast and sea weeds have already been published. Palmieri et al. (2000) compared biosorption of Nd from acidic solutions (pH of 1.5) by three biological species: a microalga (*Monoraphidium* sp.), baker’s yeast and an ascomycetous fungus (*Penicillium* sp.). The authors reported higher sorption capacities of 1511 mg*g^−1^ (10 mmol*g^−1^), 313 mg*g^−1^ (2.2 mmol*g^−1^) and 178 mg*g^−1^ (1.2 mmol*g^−1^), respectively than we found here. Tunali and Yenigun (2021) found a Q_Nd_ of about 240 mg*g^−1^ (1.7 mmol*g^−1^) for dried biomass of *Chlorella vulgaris*, and data compiled by Andrès et al. (2003) shows a Q_Nd_ of 1.1 mmol*g^−1^ and a Q_Eu_ of 0.83 mmol*g^−1^ for bacteria and yeast, hence slightly higher values but more or less in the same range as determined in this study. Kücüker et al. (2017) reported a maximum Nd uptake at pH of 5 and 35 °C of 157.4 mg*g^−1^ (1.1 mmol*g^−1^) using the biomass of *Chlorella vulgaris*. Furthermore, the examination of the binding of REEs by seaweed revealed a Q_Nd_ of 0.7 mmol*g^−1^ and a Q_Eu_ of 0.63 mmol*g^−1^, thus being in a good agreement with the data presented here (Oliveira and Garcia Jr. 2009). In good correspondence with our results, the application of phosphorylation (using cyclo-triphosphate) to dry baker’s yeast investigated by Ojima et al. (2019) resulted in an improved capacity for Nd (0.77 mmol*g^−1^), whilst it was much lower for untreated yeast (0.08 mmol*g^−1^). Interestingly, similarly to our data, also the latter two groups found slightly lower binding capacities of the investigated biomass for Eu in comparison to that for Nd. To the best of our knowledge, sorption of REEs onto the surface of a moss has not yet been described by other groups in the literature. Nevertheless, sorption capacities of Zn^2+^ onto several moss species were investigated by various researchers reporting values for Q_max, Zn_ ranging from 0.08 up to 0.93 mmol*g^−1^_dry weight (dw)_ on moss that was collected in natural habitats (Gonzalez et al. 2016, Kłos et al. 2014, Martins et al. 2004, Zhang and Banks 2005). With moss, the moss that was cloned from a single native sample and cultivated axenically under standardised conditions a Q_max, Zn_ of up to 4.6 mmol*g^−1^_dw_ was observed (Gonzalez et al. 2016).

By expanding our investigations to the biosorption of additional metals and other possibly disturbing biosorption metal ions, further interesting aspects could be observed. The overall highest Q_max_ in this work was found for gold and *P. patens* (1.59 mmol*g^−1^). The sorption capacity for Au of *P. patens* was about 2.4-fold higher compared to that of *C. brevissima* or *C. kessleri*. For comparison, Itouga et al. (2017) described the sorption capacity for Au and *Funaria hygrometrica* to be 0.6 mmol*g^−1^_dw_, corresponding well with the values determined for *C. brevissima* and *C. kessleri* in this study. Furthermore, Tunali and Yenigun (2021) also reported for sorption experiments performed at similar conditions as in this studies and with the biomass of *Chlorella vulgaris* an Au uptake of 165.5 mg*g^−1^ (0.84 mmol*g^−1^).

The second highest maximum sorption capacity, again for all three species, was observed for Pb resulting in Q_max_ of 0.8 mmol*g^−1^ for *P. patens*, 0.6 mmol*g^−1^ for *C. brevissima* and 0.4 mmol*g^−1^ for *C. kessleri*. In comparison, previous investigations regarding the biosorption of Pb(II) on various, mostly collected, moss samples report values for Q_max_, _Pb_ ranging from 0.09 mmol*g^−1^_dw_ for *Sphagnum* moss, immobilised on a polyurethane support, up to 3.6 mmol*g^−1^_dw_ for *F. hygrometrica* collected in Japan and grown on agar, thus indicating that sorption efficiencies are strongly depended on the species under investigation (Bulgariu et al. 2008, Itouga et al. 2017, Okoli et al. 2017, Zhang and Banks 2005). As with Nd and *P. patens*, the phosphorylated dry baker’s yeast mentioned above also showed very similar sorption capacity for Pb^2+^ (0.91 mmol*g^−1^) (Ojima et al. 2019), which was slightly higher than the value recorded for Nd in our work.

The comparison of the affinities of various metal ions to the here selected species revealed that Ni is the least effectively bound element. Whilst the highest capacities were registered for Au, Pb and Nd with Q_max_ values following the same order for all tested biosorbents (Q_max Au_ > Q_max Pb_ > Q_max Nd_), no such clear trends could be observed for the other metal ions. For example, Q_max_ for *P. patens* (WLE airlift cultivation) displays a sequence with Au > Pb > Nd > Pt > Eu > Fe > Cu > Ni which is different from that of *C. brevissima* Au > Pb > Nd > Cu > Eu > Pt ≈ Ni. Such differences in biomass affinities of various species for different metal ions are important and can be further used for development of biosorption-based recovery processes, for instance to successively bind target/disturbing ions by using various sorbent materials. As lead, iron, copper and nickel are the most common elements in wastewaters, either a selected biosorber should not bind them or they have to be removed prior the application of biosorption. Furthermore, the here observed differences between various affinities indicate that multiple and/or different sorption mechanisms play roles in their binding on the biomass.

### Characterisation of biosorption of REEs by the selected species

Isotherms were fitted to the data obtained for the adsorption of Nd on biomass of *P. patens*, *C. brevissima* and *C. kessleri*. The Langmuir function was suitable to describe the behaviour of the first two species, whereas the Freundlich’s isotherm was better suited in case of *C. kessleri.* The isotherms correlated well with the previous sorption experiments performed at 10 mM initial REE concentration and the same biomass. Not only was the highest sorption reached again for the moss, but the steepest initial slope of its isotherm implied efficient binding of the target element already at low concentrations. This suggests that *P. patens* biomass might be a suitable sorption material for flow-through cartridge systems for the recovery of metal ions from highly diluted aqueous solutions, thus helping to exploit alternative resources that are currently not accessible. Giese and Jordão (2019) found that the Langmuir isotherm model described the adsorption of La^3+^ on a NaOH-pretreated biomass of *Bacillus subtillis* better, whereas Tunali and Yenigun (2021) reported for *C. vulgaris* and Nd^3+^ better correlation of the experimental data with the Freundlich isotherm. According to Febrianto et al. (2009), Langmuir’s and Freundlich’s isotherms are the most abundant isotherm equations in modelling adsorption data of highly heterogeneous biological adsorbent materials. Even though these empirical models are very helpful in assessing the potential of a biosorbent, they only describe the sorption properties of this adsorber for a specific metal ion. Furthermore, they describe the net effect of all mechanisms of sorption, reduction and precipitation, that specifically occur for an ion, and do not allow any further differentiation.

The binding of Nd by *P. patens*, *C. brevissima* and *C. kessleri* from solutions with various pH (from 1 to 6) provided further differences regarding the binding properties of the biomass. Whilst for the moss comparably high sorption was observed already at pH values between 3 and 6, the binding of this REE increased linearly over the whole examined range of pH (1 to 6) for the cyanobacterium and the green alga and did not reach the level found for *P. patens.* This is in good agreement with the data published for brown algae by Vijayaraghavan et al. (2010, 2011) and Bulgariu and Bulgariu (2012). Kazak et al. (2018) tested REE sorption using several strains of heterotrophic bacteria (*Microbacterium* sp., *Curtobacterium* sp., *Bacillus subtilis*, *Pseudomonas putida*, and *Bacillus pumilis*) at pH 2 and 4 and reported in each case higher sorption coefficients at higher pH. Comparably, Tunali and Yenigun (2021) found higher sorption capacities for Nd and *C. vulgaris* at pH 5 in comparison to pH 4 and 6. Although Minoda et al. (2015) reported efficient biosorption of Nd^3+^, Dy^3+^ and La^3+^ on the biomass of *Galdieria sulphuraria* from solutions with pH between 1.5 and 2.5, lower sorption at low pH values can be explained by an increased competition between Nd and protons and displacement of the first by substantially smaller protons.

Comparing the acid-based titration curves with the reference curve (without biomass), protonation rates of 100, 79 and 67% were determined for *C. kessleri*, *C. brevissima* and *P. patens*, respectively, whilst PECs showed the reversed order (PEC _*P. patens*_ = 3.5 mmol*g^−1^, PEC _*C. brevissima*_ = 3.0 mmol*g^−1^, PEC _*C. kessleri*_ = 2.5 mmol*g^−1^). This indicates that the degree of protonation influences the metal sorption in the opposite direction. In comparison, Kiefer et al. (1997) reported PEC for *Chlamydomonas reinhardii* (green alga) and *Cyclotella cryptica* (diatom) of 1 mmol*g^−1^, thus at least 2.5-fold lower than for the species examined here. Considering the PEC and higher Q_max_ values for REEs determined in this work, the application of all three biomasses as biosorbents for REE recovery from wastewaters could be possible. In addition, a lower sorption capacity than expected due to the determined PEC indicates that the protonation rate of the biomass used may also have to be taken into account for such applications. At the same time, the Q_max_ value reported here for Au and the examined moss was higher (1.59 ± 0.07 mmol*g^−1^) than the expected based on the hypothesis of ion exchange considering trivalent gold ions (PEC_*P. patens*_/3 = 1.2 mmol*g^−1^). We thus suggest other mechanism for Au^3+^ removal from the solution. A plausible alternative is the reduction to elemental metals, previously described for gold and the biomass of the brown alga *Fucus vesiculosus* (Mata et al. 2009) or palladium and the biomass of another moss species (*Racomitrium lanuginosum*) (Sari et al. 2009).

Moreover, the pKa values found in this study for the investigated species are in good agreement with previous reports. For example, Chojnacka et al. (2005) published pK_a_ values of 6.8 to 7.8 corresponding to phosphate and hydroxyl groups and pK_a_ values of 10.8 to 11.7 for amine groups for *Spirulina* sp. Kiefer et al. (1997) reported pK_a_ values of 3.2 to 4.9 for terminal carboxy groups and pK_a_ 9.0 to 9.8 for α-amino and phenolic hydroxyl groups. Slight differences in the pKa values for the same functional groups can most probably be explained by different chemical surroundings as well as different dissociation patterns in the various matrices. Nevertheless, all these functional groups can participate in proton-to-metal-ion-exchange at different pH conditions.

In addition, carboxy and hydroxy groups, which play an exceptional role in proton-to-metal-ion-exchange, seem to appear at various pK_a_ values in the here examined species, thus pointing to different chemical surroundings and different dissociation patterns. This can in turn explain differences in sorption capacities of the three species at different pH values. Finally, most wastewater samples, where higher concentration of REEs can be expected, have pH values less than or equal to three. At this pH, all functional groups discussed here are fully protonated resulting in increased proton-to-metal-exchange abilities of the biomasses.

In summary, interesting results regarding biosorption properties of three different biological species (a moss — *P. patens*, a cyanobacterium — *C. brevissima*, and a green alga — *C. kessleri*) for REEs and other precious and possibly biosorption influencing metal ions are shown in this work. The highest binding capacity for REEs and gold was registered for *P. patens*, which makes this species particularly interesting for the application as a biosorber. However, we also report surprisingly different biosorption capacities for this species depending on the cultivation mode. Thus, further studies are needed to better assess the potential of *P. patens* as a biosorber for REEs. Moreover, the modelling of the biosorption mechanisms of Nd^3+^ to the biomass of *P. patens* should be carried out, for a better understanding of this processes and to develop practical applications. Furthermore, when considering biomass as biosorber for industrial applications not only biosorption of the target elements itself but also the productivity of the biomass in the required cultivation mode must be considered for evaluating its feasibility. Finally, kinetic studies of the selective binding of the target metal ions from multicomponent solutions have to be performed to enable a development of the intended biosorption-based recovery of the high value metal ions. Therefore, selectivity investigations must be conducted for mimicking of the binding from environmental samples and an advanced understanding of binding properties and biosorption mechanisms of selected biological species should be forced.

## Data Availability

The data that support the findings of this study are available from the corresponding author upon reasonable request.
